# Supplementary material for: Analysis of blaCHDL Genes and Insertion Sequences Related to Carbapenem Resistance in Acinetobacter baumannii Clinical Strains Isolated in Warsaw, Poland
Source: Int J Mol Sci. 2021 Mar 2;22(5):2486. doi: 10.3390/ijms22052486 (PMC7957893; doi:10.3390/ijms22052486)
Supplement: Supplementary file 1 [file ijms-22-02486-s001.pdf]

Supplementary information

"Analysis of *bla*CHDL genes and insertion sequences related to carbapenem resistance in *Acinetobacter baumannii* clinical strains isolated in Warsaw, Poland"

Alicja Słoczyńska, Matthew E. Wand, Stefan Tyski, and Agnieszka E. Laudy

Table S1. Occurrence of the acquired *bla*CHDL genes, the β-lactam susceptibility profiles and the carbapenemase activity data of whole genome sequenced *A. baumannii* isolates (n=15).

Groups of isolates carrying the acquired genes	Isolate number	MIC in mg/L (Interpretation of susceptibility results)[a]						Result of CarbAcinetoNP test (time[b])
		MEM	IPM	FEP	CAZ	PIP	PIP/TZP	
IS*Aba3*-*bla*OXA-58-like (n=2)	43	64 (R)	16 (R)	16 (I)	≥64 (R)	≥128 (R)	≥128 (R)	Uninterpretable[c] (120 min)
	52	8 (R)	16 (R)	16 (I)	≥64 (R)	≥128 (R)	≥128 (R)	Uninterpretable (120 min)
IS*Aba1*-*bla*OXA-23-like (n=7)	86	16 (R)	16 (R)	≥32 (R)	≥64 (R)	≥128 (R)	≥128 (R)	Positive (45 min)
	87	32 (R)	16 (R)	16 (I)	≥64 (R)	≥128 (R)	≥128 (R)	Positive (45 min)
	96	32 (R)	32 (R)	≥32 (R)	≥64 (R)	≥128 (R)	≥128 (R)	Positive (25 min)
	113	64 (R)	32 (R)	≥32 (R)	≥64 (R)	≥128 (R)	≥128 (R)	Positive (45 min)
	118	64 (R)	32 (R)	16 (I)	≥64 (R)	≥128 (R)	≥128 (R)	Positive (60 min)
	129	8 (R)	8 (R)	≥32 (R)	≥64 (R)	≥128 (R)	≥128 (R)	Uninterpretable (120 min)
	185	8 (R)	16 (R)	≥32 (R)	≥64 (R)	≥128 (R)	≥128 (R)	Uninterpretable (120 min)
*bla*OXA-24-like (n=6)	76	128 (R)	64 (R)	16 (I)	≥64 (R)	≥128 (R)	≥128 (R)	Positive (45 min)
	81	128 (R)	64 (R)	16 (I)	≥64 (R)	≥128 (R)	≥128 (R)	Positive (35 min)
	159	32 (R)	32 (R)	≥32 (R)	≥64 (R)	≥128 (R)	≥128 (R)	Positive (55 min)
	165	128 (R)	32 (R)	16 (I)	≥64 (R)	≥128 (R)	≥128 (R)	Positive (25 min)
	176	128 (R)	64 (R)	16 (I)	≥64 (R)	≥128 (R)	≥128 (R)	Positive (5 min)
	195	128 (R)	32 (R)	16 (I)	≥64 (R)	≥128 (R)	≥128 (R)	Positive (100 min)

R-resistant, I-intermediate, S-susceptible isolate

[a]Interpretation of MIC values of β-lactams as susceptibility of studied isolates were performed according to CLSI guidelines 2018 [1].

[b]CarbAcinetoNP test was performed as described by Dortet et al. [2]. In parentheses is the time after which the positive test was observed. The maximum incubation time for the samples was 120 min.

[c]Uninterpretable (optical reading indicated only slight change in color of phenol red solution in the test tube) results were observed. In these cases, compared to the internal control (red color tube), only the development of a red-orange color was observed in the test tube, but not the expected yellow or orange color, after the required incubation time, i.e. a maximum of 120 min.

[1]. Clinical and Laboratory Standards Institute. *Performance Standards for Antimicrobial Susceptibility Testing*. CLSI supplement M100. 28th ed. Wayne, Pennsylvania, USA. 2018

[2]. Dortet, L.; Poirel, L.; Errera, C.; Nordmann, P. CarbAcineto NP test for rapid detection of carbapenemase-producing *Acinetobacter* spp. *J Clin Microbiol* 2014, *52*, 2359-2364, doi:10.1128/JCM.00594-14
